# What Child and Adolescent Psychiatry Means to Me

**Published:** 2008

**Authors:** L. Eugene Arnold

**Affiliations:** * *Professor Emeritus of Psychiatry at Ohio State University, where he was formerly director of the division of child and adolescent psychiatry and vice-chair of psychiatry*

I have been a grandfather for only 13 years, but for 37 years I have lived a grandparent's dream: people pay me to tell them how to raise their children. This is only one of the many rewards child and adolescent psychiatry has offered me. Table 1 lists some more of them.

**Table 1:** Why I Like Being a Child and Adolescent Psychiatrist

• Growth and development heal many things that the doctor can take credit for
• Patients seldom die
• Privilege of seeing children improve and watching young success
• Acceptance into the family's emotional life
• Wide options for specialization; lots of ways to make yourself useful, which is good for the doctor's self-esteem
• Chance to be a role model, which is also good for doctor's self-esteem
• Chance to be confronted by precocious sages, which is good for doctor's humility
• Chance to witness parental heroism, which is also good for doctor's humility
• Interesting cocktail-party observations of new acquaintances when they ask your occupation (also good for the doctor's humility)
• Continual learning, whether you want it or not

Probably the greatest satisfaction in child psychiatry is the wide selection of options for specialization: psychotherapy, psychopharmacology, nutrition, biochemistry, genetics, family therapy, parent guidance, custody and visitation advice, epidemiology, disorder specialization, research, consultation, public education and best of all, the chance to integrate it all and play at being a comprehensive physician. The myriad challenges provoke learning and continued development that keep one young at heart and mind. Sometimes I think I should pay to practice child psychiatry.

It should be obvious by now to even the casual reader that I enjoy the privilege of being a child and adolescent psychiatrist and that enjoyment manifests in a playful attitude. That playful attitude includes not taking myself too seriously. In fact, I'm proud of my humility! It was earned at the expense of repeated humbling experiences in two ways:

1. Confronting tragic situations that I could not help, where all my education, training, experience and brilliant diagnostic insight seemed useless; and
2. Witnessing real heroism by some parents who struggle with sick children's difficult problems without complaining and with indefatigable hope. They outshine any professional pretensions of mine. By showing me my limitations and forcing me to compare myself to patients and parents (and occasional colleagues) of superior moral caliber, child psychiatry has made a better, more honest person of me and for this I'm grateful.

On the other hand, there is the mind-blowing exhilaration of watching a child improve after some prescription, potion or psychotherapeutic intervention and being allowed to believe that I had something to do with the improvement. The healing power of nature is truly a marvel to behold. Even more marvelous is being allowed to be a party to it, to enjoy the privilege of assisting that mysterious healing power. The experience is doubly gratifying, because in child psychiatry we have the privilege of helping two (or more) people in tandem: the child and the parent who was suffering with his/her child. It is almost as much pleasure to watch the parents’ spirits soar as the child improves, as it is to watch the child improve. I have often said that in stimulant studies of attention-deficit/hyperactivity disorder, I can break the blind by looking at the mother's face at a return appointment:…” if she looks tired and discouraged, it's placebo; if she smiles and looks happy, it's active drug.

Child psychiatry has offered a constant learning experience, not only from the rapid expansion of knowledge in the field, but from contact with children, parents, students, residents, colleagues, interdisciplinary clinical teams, multi-site research teams and most recently, from email questions from the public about an article I had not previously seen.

However, I soon learned that there are many useful things no one will teach me because no one yet knows. In trying to find new answers to unanswered questions, I was seduced into research and now enjoy child psychiatry even more. In clinical treatment research, I can enjoy all the advantages of clinical practice (without the need for insurance paperwork) and in addition experience the thrill of data analysis. Analyzing the data is like opening a surprise package, like unwrapping a Christmas present: you don't know what you're getting; you may be hoping for a certain thing, but have no assurance that's what you will get. Sometimes it's a lump of coal, but even that you can use to light the fire for the next study.

The fact that we are dealing with growing, developing organisms should direct our attention to the building blocks for that growth/development: nutrients. One of my pleasures has been to participate in research on nutritional aspects of child psychiatry. Equally gratifying is to participate in efforts to make such interest and research respectable. The implicit double standard about drugs and psychosocial treatments on the one hand and nutrition and other complementary considerations on the other seems to make many intelligent colleagues reluctant to acknowledge any interest in this area. The discovery of closet nutritionists after boldly speaking out on the subject has been a repeated joy.

The experience of observing nature's healing power, coupled with the interest in research, has led me to a great interest in placebo/Hawthorne effect. I'm fascinated by the fact that this no-risk treatment can produce an improvement, depending on the disorder, that is a tenth to twice as much as active medication. For example, if an antidepressant has a 60% response rate and placebo 40% response rate, then the pharmacological effect is 20% response, the difference. In this case, the placebo effect is twice as strong as the pharmacological effect and when you give an antidepressant, 2/3 of the effect is placebo. This is why witch doctors, shamans and charlatans thrive: in the example given, they can be 2/3 as effective as the most expert allopathic psychiatrist. We need to understand this effect better: how much of it is real improvement, how much statistical regression to the mean, how much rater expectation, etc.? Mastery of placebo effect for the good of our patients is particularly important in child psychiatry because children are subject to two sets of expectations: their own and their parents’ and they are probably more sensitive to the expectation matrix than adults are. We should strive to be good witch doctors as well as good psychotherapy technicians, pharmacologists and nutrition experts. I can proudly (and humbly) say that I've become the best witch doctor I can be.

And that's what child and adolescent psychiatry means to me.

## About the Author

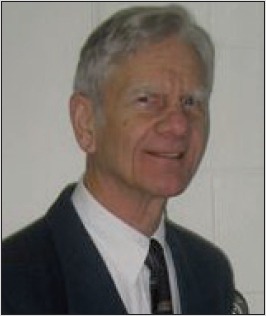



L. Eugene Arnold, M.Ed, M.D. is Professor Emeritus of Psychiatry at Ohio State University, where he was formerly director of the division of child and adolescent psychiatry and vice-chair of psychiatry. He graduated from Ohio State University College of Medicine magna cum laude, interned at University of Oregon, took residencies at Johns Hopkins, where he earned the M.Ed. and served in the U.S. Public Health Service. He is a co-investigator in the OSU Research Unit on Pediatric Psychopharmacology. He has over 37 years’ experience in child psychiatric research, including the multi-site NIMH Multimodal Treatment Study of Children with ADHD (“the MTA”), for which he continues as executive secretary and current chair of the steering committee. A particular interest is alternative and complementary treatments for ADHD. His publications include 9 books, 55+ chapters and 150+ articles.

